# A UiO-66-NH_2_ MOF/PAMAM Dendrimer Nanocomposite for Electrochemical Detection of Tramadol in the Presence of Acetaminophen in Pharmaceutical Formulations

**DOI:** 10.3390/bios13050514

**Published:** 2023-04-30

**Authors:** Fariba Garkani Nejad, Hadi Beitollahi, Iran Sheikhshoaie

**Affiliations:** 1 Department of Chemistry, Faculty of Science, Shahid Bahonar University of Kerman, Kerman 76175-133, Iran; 2 Environment Department, Institute of Science and High Technology and Environmental Sciences, Graduate University of Advanced Technology, Kerman P.O. Box 76318-85356, Iran

**Keywords:** electrochemical sensor, tramadol, acetaminophen, UiO-66-NH_2_ metal–organic framework, poly(amidoamine) dendrimer, glassy carbon electrode

## Abstract

In this work, we prepared a novel electrochemical sensor for the detection of tramadol based on a UiO-66-NH_2_ metal–organic framework (UiO-66-NH_2_ MOF)/third-generation poly(amidoamine) dendrimer (G3-PAMAM dendrimer) nanocomposite drop-cast onto a glassy carbon electrode (GCE) surface. After the synthesis of the nanocomposite, the functionalization of the UiO-66-NH_2_ MOF by G3-PAMAM was confirmed by various techniques including X-ray diffraction (XRD), energy-dispersive X-ray spectroscopy (EDS), field emission-scanning electron microscopy (FE-SEM), and Fourier transform infrared (FT-IR) spectroscopy. The UiO-66-NH_2_ MOF/PAMAM-modified GCE exhibited commendable electrocatalytic performance toward the tramadol oxidation owing to the integration of the UiO-66-NH_2_ MOF with the PAMAM dendrimer. According to differential pulse voltammetry (DPV), it was possible to detect tramadol under optimized circumstances in a broad concentration range (0.5 μM–500.0 μM) and a narrow limit of detection (0.2 μM). In addition, the stability, repeatability, and reproducibility of the presented UiO-66-NH_2_ MOF/PAMAM/GCE sensor were also studied. The sensor also possessed an acceptable catalytic behavior for the tramadol determination in the co-existence of acetaminophen, with the separated oxidation potential of ΔE = 410 mV. Finally, the UiO-66-NH_2_ MOF/PAMAM-modified GCE exhibited satisfactory practical ability in pharmaceutical formulations (tramadol tablets and acetaminophen tablets).

## 1. Introduction

Acetaminophen (N-acetyl-p-aminophenol), also referred to as paracetamol, is an analgesic and antipyretic medication as well as a key ingredient in some flu and cold treatments. Acetaminophen is functionally similar to aspirin, and is therefore considered a suitable alternative for aspirin-sensitive people. Combining acetaminophen with non-steroidal anti-inflammatory drugs (NSAIDs) and opioids is reportedly an effective strategy for treating pain such as post-operative or cancer pain [1,2,3]. The metabolism of acetaminophen occurs mainly in the liver, resulting in the production of toxic metabolites. Acetaminophen overdose is associated with accumulated toxic metabolites leading to the development of nephrotoxicity, hepatotoxicity, and sometimes kidney failure. Other side effects have been reported to be liver problems, pancreatitis, and skin rashes. Such bottlenecks may be due to high doses, chronic abuse, or concurrent abuse with alcohol or other agents [4,5,6].

Tramadol, (1RS, 2RS)-2-[(dimethylamine)-methyl]-1-(3-methoxy phenyl)-cyclohexanol hydrochloride), is a centrally serving synthetic analgesic with poor affinity for μ-opioid receptors. It is prescribed to manage moderate-to-severe pain and has monoaminergic performance to impede the reabsorption of serotonin and noradrenaline. Tramadol alone or in combination with NSAIDs can be prescribed for people with chronic pain, spinal cord injury, depression, postsurgical pain, and back pain [7,8,9]. Similarly to morphine, tramadol attaches to brain receptors (opioid receptors) for the reuptake of serotonin and norepinephrine, resulting in analgesic impacts. Although tramadol is an anti-addictive drug, the strong dependence of addicts on it can be attributed to its influence on opioid receptors in the central nervous system (CNS). Tramadol is not recommended at all for people who suffer from severe asthma, breathing conditions, and stomach obstruction. Tramadol overdose can cause vomiting, problems in the CNS and respiratory system, dizziness, seizures, depression, nausea, tachycardia, and coma [10,11,12].

The combination of acetaminophen and tramadol is usually recommended in pain management due to its high safety and efficacy [13]. Therefore, researchers have always been trying to determine the concentrations of these drugs, alone and in combination, in order to prevent overdose and so inhibit their toxic impacts.

Some of the methods used for the simultaneous determination of these analytes are high performance liquid chromatography [14], liquid chromatography coupled with mass spectrometry [15], gas chromatography/mass spectrometry [16], and capillary electrophoresis [17]. Despite the advantages of these techniques, they suffer from disadvantages such as a long process, high cost, and the need for a pretreatment step. Accordingly, scientists seek to develop a sensitive, facile, and selective analytical technique for sensing these analytes.

Electrochemical sensors can selectively detect an analyte in complex matrices, which are considered tools with technical simplicity, excellent sensitivity and great adaptability for in situ determination, adjustable, and relatively inexpensive [18,19,20,21,22,23]. Despite these advantages, they are hampered by their low-speed electrode kinetics and great over-potential, which lead to less charge transfer and more interference than other electrically active samples, which co-exist with the specimen. This ultimately reduces the sensing capacity of the instruments with respect to the material [24,25].

This problem can be circumvented with the help of sensor materials with great conductivity, long stability, and appropriate catalytic performance. The surface of the electrode can be modified with modifiers, which in this case can increase the speed of electron transfer and current sensitivity, and minimize over-potential. Hence, many efforts have been made recently to find a modified electrode material for sensitive and selective detection [26,27,28,29].

In the meantime, outstanding attention has been directed toward nanomaterials that have shown a wide range of practical applications [30,31,32,33,34,35,36,37,38,39]. Nanomaterials can increase the electrode surface area and the electron transfer between the modified surface and redox centers of the targeted molecules [40,41,42,43,44,45]. The incorporation of versatile nanomaterials with electrodes can enhance the properties of sensors, and enable reliable and sensitive quantification of various biomolecules at clinical levels [46,47].

Dendrimers are nano-sized and monodispersed synthetic polymers showing a highly branched three-dimensional regular structure. The poly(amidoamine) (PAMAM) dendrimer has unique properties, some of which are of a high physical and chemical stability, biocompatibility, structural flexibility, excellent monodispersity, tunable porosity, and high performance. Due to a myriad of amine groups, PAMAM dendrimers act as promising platforms for the fabrication of electrochemical sensors applicable in biomedicine, gene therapy, and the catalysis industry [48,49]. In addition, the synergistic effects of dendrimer composites would further broaden their application in the field of electrochemical sensors [50].

Metal-organic frameworks (MOFs) are made by self-assembly of metal ions associated with organic ligands. Some of the excellent properties of MOFs include high porosity, ordered crystal structures, large surface areas, and tunability for pore size and high performance. Metal ions and organic linkers of well-designed MOFs have been used to construct new electrochemical sensors [51,52,53,54,55,56,57,58,59]. Zr-based MOFs have diverse structures, low toxicity, remarkable stability, and especially unique thermostability [60,61]. The MOFs enhance the conductivity of the dendrimer nanocomposites, thus resulting in a synergistic impact to enhance the electrochemically active area and the sensitivity of the electrochemical sensor.

The current work was conducted to produce and characterize the UiO-66-NH_2_/G3-PAMAM nanocomposite, which was then anchored on a GCE to achieve an electrocatalytic and voltammetric tramadol sensor. The sensor based on the UiO-66-NH_2_ MOF/PAMAM nanocomposite demonstrated a high degree of catalysis toward tramadol, with a narrow LOD and a wide linear range. Additionally, the sensor was tested in terms of catalytic behavior for the tramadol determination in the co-existence of acetaminophen. The ability of the modified electrode for sensor applications was examined in real specimens of acetaminophen tablets and tramadol tablets.

The novelty of this research lies in the combination of the UiO-66-NH_2_ MOF and the PAMAM dendrimer as a sensing platform, which has enabled the detection of tramadol in the presence of acetaminophen.

## 2. Experimental

### 2.1. Instruments and Reagents

An Autolab PGSTAT302N electrochemical device was employed to conduct electrochemical determinations. The solutions’ pHs were adjusted by a pH-meter (Metrohm 710). The deionized water used in each experiment was also taken from a Millipore Direct-Q^®^ 8 UV (ultra-violet) (Millipore, Darmstadt, Germany).

Field-emission scanning electron microscopy (MIRA3TESCAN-XMU) was utilized for morphological studies. Energy-dispersive X-ray spectroscopy (EDS)-FE-SEM (MIRA3TESCAN-XMU, Tescan, Czech Republic) was also applied for elemental analysis. XRD patterns were recorded to obtain the data on structure using a Panalytical X’Pert Pro X-ray diffractometer (Etten Leur, The Netherlands) via Cu/Kα radiation (λ = 1.5418 nm). The FT-IR spectra were also obtained through a Bruker Tensor II spectrometer (Mannheim, Germany).

The precursors for the synthesis of the UiO-66-NH_2_/G3-PAMAM nanocomposite, tramadol, acetaminophen, and other chemicals were also of analytical grade and were used upon delivery without any additional purification. It is noted that they were obtained from Merck and Sigma-Aldrich chemical companies.

### 2.2. Synthesis of the UiO-66-NH_2_ MOF/G3-PAMAM Nanocomposite

The previous report, with slight modification, was followed to construct the UiO-66-NH_2_ MOF [62]. Thus, ZrCl_4_, 2-aminoterephthalic acid, and glacial acetic acid (0.2 mmol, 0.2 mmol, and 5 mL, respectively) were poured into dimethylformamide (20 mL, DMF) while ultra-sonicating for 45 min, followed by their placement in a 50 mL Teflon-lined autoclave and subsequently in the oven at 120 °C for 48 h. After cooling down, a centrifugation was conducted to separate the precipitate, followed by rinsing thoroughly with DMF/methanol and then vacuum-drying at 70 °C for 12h to obtain the UiO-66-NH_2_ MOF.

Our previous report described the preparation steps and characteristics of G3-PAMAM [63]. Thus, the UiO-66-NH_2_ MOF/G3-PAMAM nanocomposite was prepared by appending the UiO-66-NH_2_ MOF (50 mg) to methanol (25 mL) containing 5% glutaraldehyde, followed by stirring and heating at 40 °C for 2 h. Then, the glutaraldehyde-modified UiO-66-NH_2_ was rinsed three times with methanol. Next, G3-PAMAM (0.025 mmol) was poured into methanol (50 mL) containing UiO-66-NH_2_/glutaraldehyde while stirring at room temperature for 3 h. Finally, the obtained UiO-66-NH_2_ MOF/PAMAM nanocomposite was rinsed with methanol and vacuum-dried at 50 °C.

### 2.3. Preparation of GCE Modified with the UiO-66-NH_2_ MOF/G3-PAMAM Nanocomposite

A facile drop-casting protocol was followed to modify the surface of GCE with the UiO-66-NH_2_ MOF/G3-PAMAM nanocomposite. Thus, a 1 mg/mL suspension of prepared nanocomposite underwent a 20-min sonication, and then 4 μL of the suspension was poured on the GCE surface in a dropwise manner, followed by drying under room conditions to achieve the UiO-66-NH_2_ MOF/PAMAM/GCE.

### 2.4. Preparation of Pharmaceutical Formulations

Five tablets of the tramadol (labeled value of tramadol = 100 mg per tablet) and acetaminophen (labeled value of acetaminophen = 325 mg per tablet) purchased from a local pharmacy in Kerman (Iran) were completely powdered in a mortar and pestle. Then, an accurately weighed amount of the homogenized tramadol and acetaminophen powders was transferred into 100 mL 0.1 mol/L PBS (pH 7.0). For better dissolution, the solutions inside the flasks were sonicated (20 min). After that, the resulting samples were filtered. Finally, a specific volume of the prepared samples was transferred into volumetric flasks and diluted with 0.1 M PBS (pH = 7.0). The diluted solutions were then put into the electrochemical cell for DPV analysis.

## 3. Results and Discussion

### 3.1. Characterization of the UiO-66-NH_2_ MOF/PAMAM Nanocomposite

**Figure 1:**
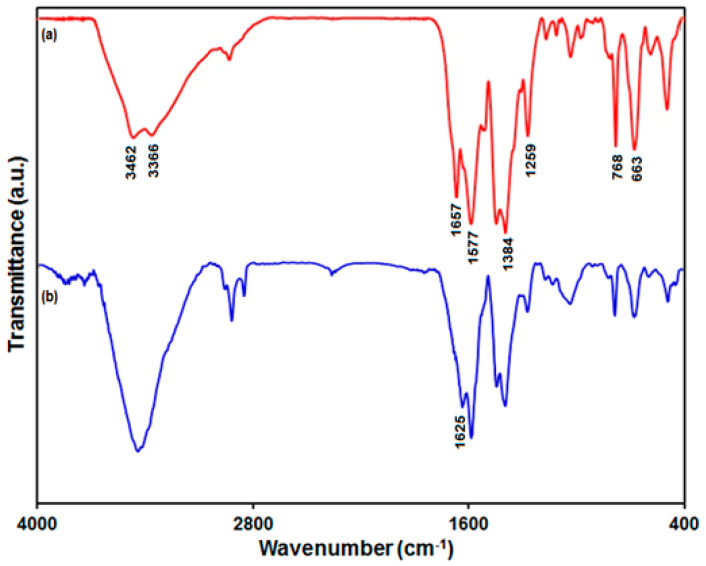
FT-IR spectrum of UiO-66-NH_2_ MOF (a) and UiO-66-NH_2_ MOF/PAMAM nanocomposite (b).

FT-IR spectroscopy authenticated that the PAMAM cross-linked on the UiO-66-NH_2_ MOF (Figure 1). The characteristic peaks of the FT-IR spectrum prepared from the UiO-66-NH_2_ MOF at 1577 cm^−1^ and 1657 cm^−1^ were attributed to the stretching vibration of C=C on the benzene ring and the vibration of coordinated carboxylate moieties, respectively [64]. The bonds at 1384 cm^−1^ and 1259 cm^−1^ were attributed to the stretching vibrations of C–N related to 2-aminoterephthalic acid. The characteristic peaks at 3366 cm^−1^ and 3462 cm^−1^ were attributed to symmetric and asymmetric stretching vibrations of N-H related to the primary amine group, respectively. The characteristic peaks at 768 cm^−1^ and 663 cm^−1^ were attributed to the vibration of Zr–O on the UiO-66-NH_2_ [65]. In the FT-IR spectrum of the UiO-66-NH_2_ MOF/PAMAM nanocomposite, the observed absorption peak at 1657 cm^−1^ of UiO-66-NH_2_ shifted to 1625 cm^−1^ following PAMAM immobilization, authenticating the formation of imine bands (C=N) from a Schiff base reaction between the carbonyl groups, belonging to glutaraldehyde, and the amine group, belonging to the UiO-66-NH_2_ MOF and the PAMAM dendrimer.

**Figure 2:**
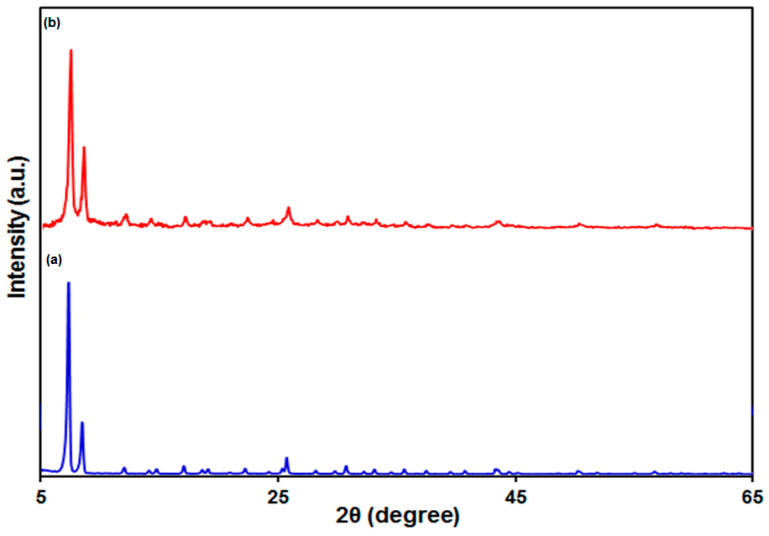
The XRD pattern of UiO-66-NH_2_ MOF (a) and UiO-66-NH_2_ MOF/PAMAM nanocomposite (b).

Figure 2a shows the XRD pattern verifying crystallinity of the as-produced UiO-66-NH_2_ MOF. As seen, the characteristic peaks generated from the UiO-66-NH_2_ MOF were in line with earlier reports [60,66]. The characteristic peaks of the UiO-66-NH_2_ MOF/PAMAM nanocomposite were the same as those of the UiO-66-NH_2_ MOF (Figure 2b)*,* which means that this sample did not change in crystal structure following modification with PAMAM.

**Figure 3:**
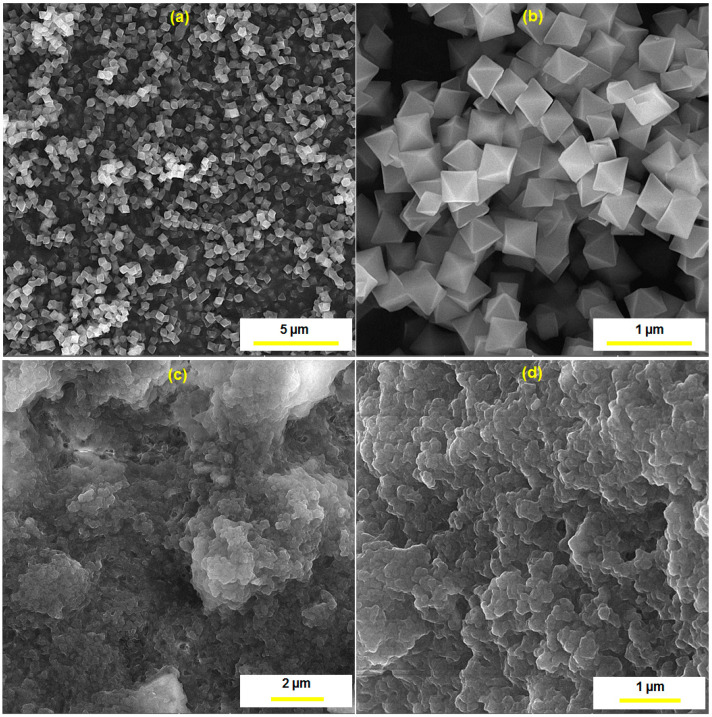
The FE-SEM images of the as-synthesized UiO-66-NH_2_ MOF (**a**,**b**) and UiO-66-NH_2_ MOF/PAMAM nanocomposite (**c**,**d**) at different magnifications.

Figure 3 illustrates the FE-SEM images prepared from the UiO-66-NH_2_ MOF and the UiO-66-NH_2_ MOF/PAMAM nanocomposite for morphological studies. As seen (Figure 3a,b), the UiO-66-NH_2_ sample displayed octahedral morphology with proper crystallinity. The UiO-66-NH_2_ MOF had a particle size of ~275 nm. When PAMAM was loaded onto the UiO-66-NH_2_ MOF, the octahedral edges and corners of the UiO-66-NH_2_ MOF were destroyed and its surface was coated with the PAMAM dendrimer (Figure 3c,d).

**Figure 4:**
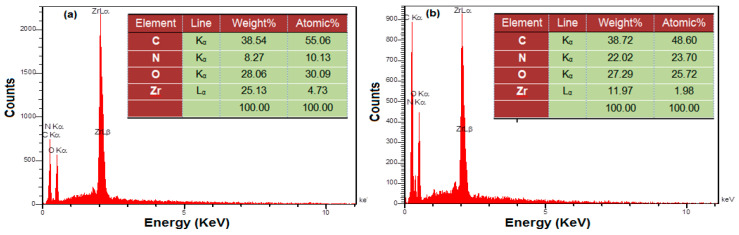
EDS analysis of UiO-66-NH_2_ MOF (**a**) and UiO-66-NH_2_ MOF/PAMAM nanocomposite (**b**).

The EDS was recruited to carry out the elemental analyses (Figure 4). As seen, the main peaks in spectrum 4a exhibited the elements Zr, C, N, and O, which indicates the chemical purity of the as-constructed UiO-66-NH_2_ MOF. Figure 4b depicts the EDS spectrum acquired from the UiO-66-NH_2_ MOF/PAMAM nanocomposite. PAMAM loading on the UiO-66-NH_2_ MOF caused an increase in the level of N element (8.27–22.02% by weight).

### 3.2. Investigating the Effect of the UiO-66-NH_2_ MOF/PAMAM Nanocomposite on the Electrochemical Behavior of Tramadol

The tramadol electro-oxidation has an association with electron and proton exchange. Thus, the effect of pH on the electrochemical response of the tramadol on the UiO-66-NH_2_ MOF/PAMAM-modified GCE sensor should be evaluated. For this purpose, experiments were performed in 0.1 M PBS at the pH range of 2.0–9.0 using DPV. The results showed more oxidation of tramadol on the surface of the UiO-66-NH_2_ MOF/PAMAM-modified GCE in neutral conditions relative to acidic or alkaline conditions, so pH 7.0 was considered as the optimal value for the electro-oxidation of tramadol on the as-produced electrode surface.

**Figure 5:**
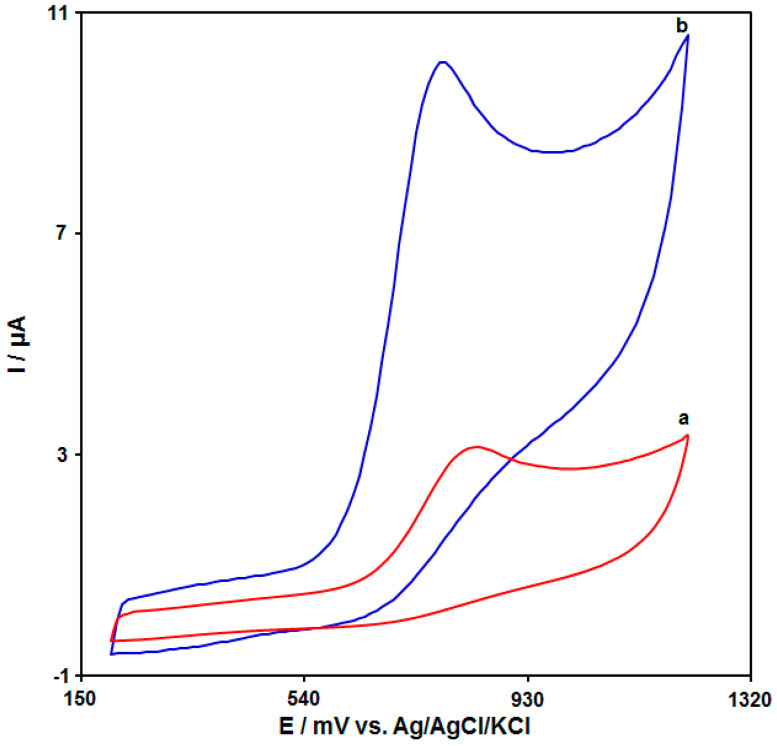
CVs acquired for (a) bare GCE and (b) UiO-66-NH_2_ MOF/PAMAM-modified GCE for tramadol (100.0 μM) in PBS (pH 7.0, 0.1 M) at a scan rate of 50 mV/s.

The cyclic voltammograms (CVs) were acquired for the tramadol solution (100.0 μM) in PBS (pH 7.0, 0.1 M) on the bare GCE (Figure 5 (curve a)) and the UiO-66-NH_2_ MOF/PAMAM-modified GCE (Figure 5 (curve b)). The tramadol oxidation current on the bare GCE surface was estimated to be 3.14 μA. There was an increase in the tramadol oxidation current on the UiO-66-NH_2_ MOF/PAMAM-modified GCE, with a maximum value of about 10.1 μA. Hence, the combination of the UiO-66-NH_2_ MOF and PAMAM dendrimers can greatly enhance the detection sensitivity.

### 3.3. The Scan Rate Effect on the Oxidation of Tramadol on the UiO-66-NH_2_ MOF/PAMAM-Modified GCE

**Figure 6:**
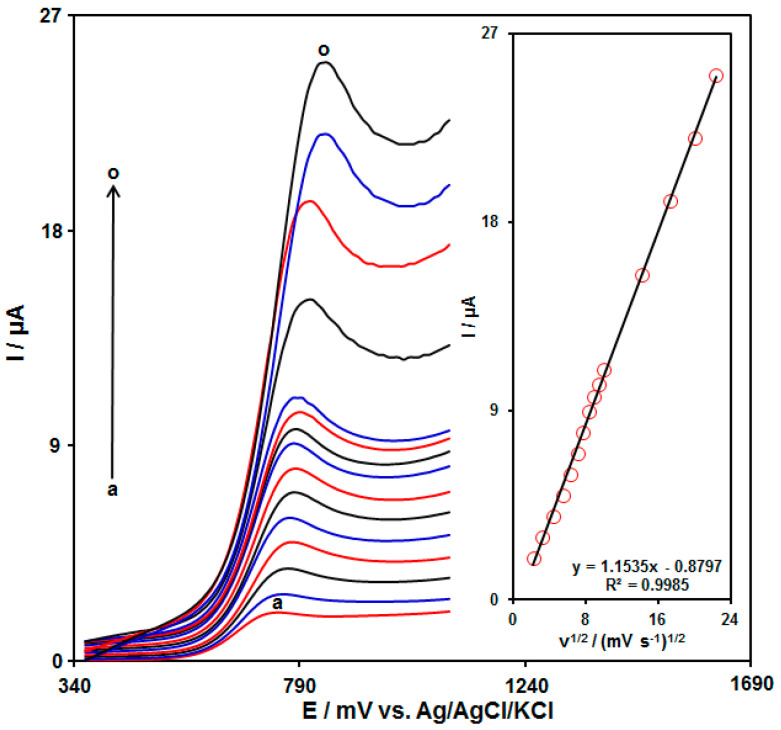
LSVs acquired for tramadol (70.0 μM) in PBS (pH 7.0, 0.1 M) on UiO-66-NH_2_ MOF/PAMAM-modified GCE at variable scan rates ((a: 5 mV/s), (b: 10 mV/s), (c: 20 mV/s), (d: 30 mV/s), (e: 40 mV/s), (f: 50 mV/s), (g: 60 mV/s), (h: 70 mV/s), (i: 80 mV/s),(j: 90 mV/s), (k: 100 mV/s), (l: 200 mV/s), (m: 300 mV/s), (n: 400 mV/s), and (o: 500 mV/s)). Inset: Plot of Ipa against υ^1/2^ for tramadol electro-oxidation.

The electrochemical response of tramadol on the UiO-66-NH_2_ MOF/PAMAM-modified GCE was evaluated by the linear sweep voltammetry (LSV) method. Figure 6 shows the LSVs acquired for tramadol (70.0 μM) on the UiO-66-NH_2_ MOF/PAMAM-modified GCE in PBS (pH = 7.0, 0.1 M) at variable scan rates. The LSVs show an increase in the oxidation peak currents with enhanced applied scan rates. Figure 6 (inset) depicts the anodic peak current (Ipa) of tramadol, representing a linear relationship with the scan rate square root (υ^1/2^), Ipa = 1.1535υ^1/2^ − 0.8797 (R^2^ = 0.9985). Hence, the electrochemical behavior of tramadol follows a diffusion-controlled process.

### 3.4. Chronoamperometric Determinations

**Figure 7:**
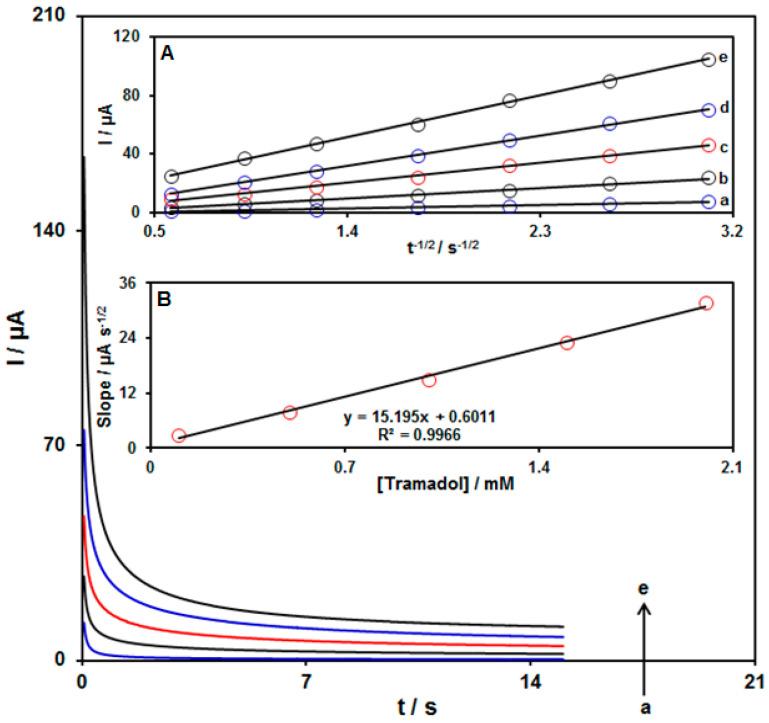
Chronoamperograms acquired for the UiO-66-NH_2_ MOF/PAMAM-modified GCE in the exposure to variable tramadol concentrations ((a: 0.1 mM), (b: 0.5 mM), (c: 1.0 mM), (d: 1.5 mM), and (e: 2.0 mM) of tramadol) in PBS (pH 7.0, 0.1 M). Inset **A**: Plots of I vs. t^−1/2^ from chronoamperograms a–e, and Inset **B**: Plot of straight line slope vs. tramadol level.

The chronoamperometry was used to explore the tramadol oxidation on the UiO-66-NH_2_ MOF/PAMAM-modified GCE surface (Figure 7). Chronoamperometric determinations for variable tramadol levels on the modified electrode were carried out at the working electrode potential of 825 mV. The diffusion coefficient (D) was measured for tramadol in aqueous solution in accordance with the Cottrell equation:

I = n F A C_b_ D^1/2^ π^−1/2^ t^−1/2^ (1)

Herein, C_b_ stands for the concentration, D for the diffusion coefficient, and A for the electrode area. Figure 7A shows the plots of I against t^−1/2^ for variable tramadol content. Figure 7B indicates the slopes from the straight lines plotted against tramadol levels. The D value was computed to be 7.9 × 10^−5^ cm^2^/s according to the slope of obtained plots and also the Cottrell equation. The D value in this work is comparable with the results reported in the literature (1.05 × 10^−5^ cm^2^/s [67], 9.2 × 10^−6^ cm^2^/s [68], 2.39 × 10^−5^ cm^2^/s [69].

### 3.5. Quantitative Determination of Tramadol by the DPV Method

**Figure 8:**
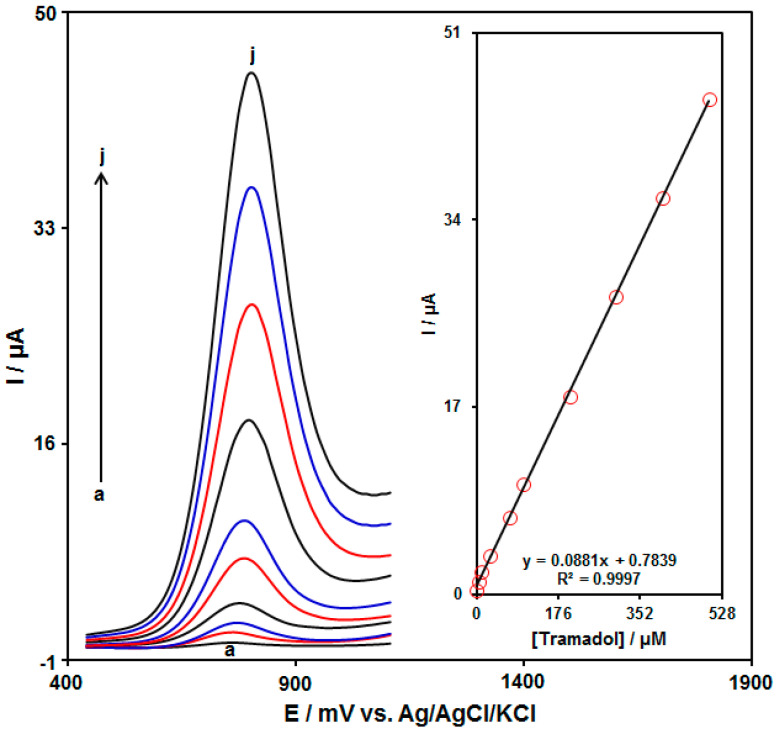
DPVs acquired for the UiO-66-NH_2_ MOF/PAMAM-modified GCE in the exposure to variable tramadol concentrations ((a: 0.5 μM), (b: 5.0 µM), (c: 10.0 µM), (d: 30.0 µM), (e: 70.0 µM), (f: 100.0 µM), (g: 200.0 µM), (h: 300.0 µM), (i: 400.0 µM), and (j: 500.0 μM)) tramadol in PBS (pH 7.0, 0.1 M). Insets: Relationships between the Ipa and levels of tramadol.

The quantitative determination of tramadol was performed using the DPV method. Figure 8 illustrates the DPVs acquired for the UiO-66-NH_2_ MOF/PAMAM-modified GCE in the exposure to variable tramadol levels. An elevation in the concentration of tramadol obviously resulted in an increase in the Ipa of tramadol. The calibration curve for variable tramadol levels revealed a linear dynamic range as broad as 0.5 μM to 500.0 μM, with the equation of Ipa = 0.0881C_tramadol_ + 0.7839 (R^2^ = 0.9997) (Figure 8, Inset). The sensitivity and LOD were calculated to be 0.0881 μM/μA and 0.2 μM for the UiO-66-NH_2_ MOF/PAMAM-modified GCE in sensing tramadol, respectively. A comparison of tramadol detection using various sensors is presented in Table 1.

### 3.6. Quantitative Determination of Tramadol in the Presence of Acetaminophen

**Figure 9:**
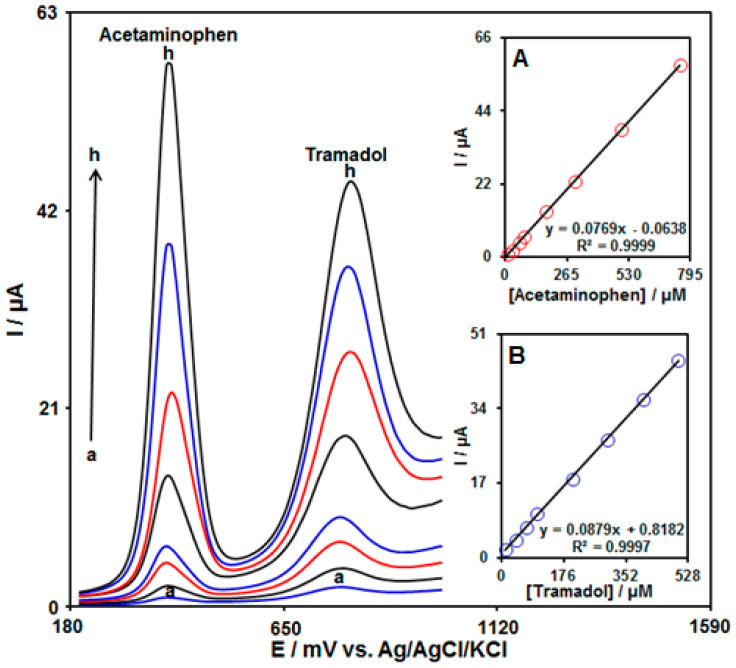
DPVs acquired for UiO-66-NH_2_ MOF/PAMAM-modified GCE in PBS (pH 7.0, 0.1 M) with variable tramadol and acetaminophen levels ((a: 10.0 μM), (b: 40.0 μM), (c: 70.0 μM), (d: 100.0 μM), (e: 200.0 μM), (f: 300.0 μM), (g: 400.0 μM), and (h: 500.0 μM) of tramadol) and ((a: 10.0 μM), (b: 30.0 μM), (c: 60.0 μM), (d: 80.0 μM), (e: 175.0 μM), (f: 300.0 μM), (g: 500.0 μM), and (h: 750.0 μM) of acetaminophen). Insets: (**A**) Plot of peak current as a function of acetaminophen levels; (**B**) Plot of peak current as a function of tramadol levels.

The current work aimed to fabricate a modified electrode capable of distinguishing the tramadol and acetaminophen at the same time. The analytical tests were performed by varying the contents of tramadol and acetaminophen at the UiO-66-NH_2_ MOF/PAMAM-modified GCE as the working electrode in PBS (pH 7.0, 0.1 M). The DPVs were acquired for the UiO-66-NH_2_ MOF/PAMAM-modified GCE at variable levels of tramadol and acetaminophen (Figure 9). Separate oxidation signals appeared at the potentials of about 780 mV and 370 mV, which correspond to the oxidation of tramadol and acetaminophen, respectively. The sensitivity of the modified electrode relative to tramadol in the absence (0.0881 µA/µM, Figure 8) and presence (0.0879 µA/µM, Figure 9B) of acetaminophen was very similar, suggesting independent oxidation of tramadol and acetaminophen on the UiO-66-NH_2_ MOF/PAMAM-modified GCE, and also the feasibility of simultaneous determinations of the two analytes with no interference.

### 3.7. The Stability, Repeatability, and Reproducibility Studies of the UiO-66-NH_2_ MOF/PAMAM-Modified GCE for Tramadol Analysis

For practical applications, the repeatability, reproducibility, and stability of the electrochemical sensors are essential. The stability of the UiO-66-NH_2_ MOF/PAMAM-modified GCE was examined by storing the electrode at laboratory temperatures. Then, the electrode was used for the analysis of 60.0 µM of tramadol from 1 to 15 day intervals in 0.1 M PBS. According to the obtained results, the UiO-66-NH_2_ MOF/PAMAM-modified GCE sensor presented only a 3.9% variation after 15 days

The repeatability of the response of the modified electrode (UiO-66-NH_2_ MOF/PAMAM-modified GCE) was estimated by performing the electrochemical experiment repeatedly (five measurements) with the same UiO-66-NH_2_ MOF/PAMAM-modified GCE sensor in a buffer solution (0.1 M, PBS) containing 60.0 µM of tramadol. The relative standard deviation (RSD) based on five replicates was found to be 4.1%, which indicated that the UiO-66-NH_2_ MOF/PAMAM-modified GCE has good repeatability.

The reproducibility of the prepared sensor was also evaluated by preparing five modified electrodes (UiO-66-NH_2_ MOF/PAMAM-modified GCE) using the same fabrication procedure. The RSD value for the peak currents obtained for these electrodes in a buffer solution (0.1 M, PBS) containing 60.0 µM of tramadol was calculated to be 2.8%, which revealed a very good reproducibility of the electrode preparation procedure.

### 3.8. The Selectivity of the UiO-66-NH_2_ MOF-PAMAM/GCE for the Detection of Tramadol

To evaluate the selectivity of the UiO-66-NH_2_ MOF-PAMAM/GCE for tramadol, an investigation into the influence of potential interfering substances was performed under the optimized conditions. The DPV responses upon addition of interfering substances into 0.1 M PBS (pH 7.0) containing 50.0 µM tramadol were recorded. The obtained results revealed no significant changes in the current of tramadol in the presence of the interfering substances (1000-fold excess of Na^+^, Mg^2+^, Ca^2+^, NH_4_^+^, SO_4_^2−^, 500-fold excess starch, fructose, glucose, lactose, sucrose, L-lysine, L-serine, 100-fold excess dopamine, uric acid, epinephrine, and norepinephrine). However, ascorbic acid showed serious interference in the tramadol determination in equal concentration.

### 3.9. Application of the UiO-66-NH_2_ MOF/PAMAM-Modified GCE Sensor for the Analysis of Acetaminophen and Tramadol in Pharmaceutical Formulations

The developed method can be used successfully for the determination of tramadol and acetaminophen in pharmaceutical formulations (tramadol tablets and acetaminophen tablets specimens). By using the standard addition method, this study was accomplished and the results of the analysis are shown in Table 2. The appreciable recovery rates (96.7–103.5%) confirmed the capability of the UiO-66-NH_2_ MOF/PAMAM-modified GCE as a voltammetric sensor for the analysis of these two drugs in pharmaceutical formulations.

## 4. Conclusions

An attempt was made to produce a tramadol sensor through the modification of GCE surface with a UiO-66-NH_2_ MOF/G3-PAMAM dendrimer. The sensor (a UiO-66-NH_2_ MOF/PAMAM-modified GCE) exhibited commendable catalytic performance toward the tramadol oxidation. There was a linear relationship between the DPV response of the modified electrode and the tramadol contents (0.5 μM–500.0 μM). The LOD was calculated at 0.2 μM. The sensor also possessed an acceptable catalytic behavior for the tramadol determination in the co-existence of acetaminophen. In addition, it was found that the UiO-66-NH_2_ MOF/PAMAM-modified GCE demonstrated good stability, repeatability, and reproducibility toward the detection of the analgesic drug, tramadol. The ability of the modified electrode for sensor applications was confirmed in specimens of acetaminophen tablets and tramadol tablets, with acceptable recovery rates.

## Figures and Tables

**Table 1:** Comparison of different sensors for tramadol detection.

Electrochemical Sensor	Electrochemical Technique	Linear Range	Limit of Detection	Sample Type	Ref.
Poly(Nile blue)/glassy carbon electrode	DPV	1–310 μM	0.5 µM	Ultracet^®^ tablets	[25]
La^3+^/ZnO nano-flowers and multi-walled carbon nanotubes/screen-printed electrode	DPV	0.5–800.0 μM	0.08 μM	Tramadol tablets and urine	[68]
Multi-walled carbon nanotubes/glassy carbon electrode	DPV	2–300 μM	0.361 µM	Human serum, urine, and ZAFIN^®^ tablets	[70]
Pt-Pd bimetallic nanoparticles/poly(diallyldimethylammonium chloride)/nitrogen-doped graphene/glassy carbon electrode	^1^ SWV	12.0–240.0 µM	5.7 µM	Infected urine	[71]
Magneto layer double hydroxide (LDH)/Fe_3_O_4_/glassy carbon electrode	DPV	1.0–200.0 µM	0.3 µM	Human serum and human urine	[72]
Graphitic carbon nitride/Fe_3_O_4_ nanocomposite/carbon paste electrode	DPV	0.2–14.0 µM and 14.0–120.0 µM	0.1 µM	Human blood serum, human blood plasma, and urine	[73]
Au nanoparticles/cysteic acid/glassy carbon electrode	SWV	0.5–63.5 µM	0.17 µM	Human blood plasma	[74]
Electrospun carbon nanofibers/screen-printed electrode	SWV	0.05–1.0 nM and 1.0–100 nM	0.016 nM	Urine	[75]
Nafion-coated tetrahedral amorphous carbon electrode	DPV	1–12.5 µM	131 nM	Human plasma	[76]
Graphene/Co_3_O_4_ nanocomposite/screen-printed electrode	DPV	0.1–500.0 μM	0.03 μM	Tramadol tablets, Acetaminophen tablets, and urine	[77]
FeNi_3_ nanoalloy/glassy carbon electrode	DPV	0.1–900.0 μM	8.2 nM	Ultracet^®^ tablets, tramadol tablets, acetaminophen tablets, serum, and urine	[78]
UiO-66-NH_2_ MOF-PAMAM/GCE	DPV	0.5–500.0 µM	0.2 µM	Tramadol tablets	This work

^1^—Square wave voltammetry.

**Table 2:** Determination of tramadol and acetaminophen drugs in pharmaceutical formulations using the UiO-66-NH_2_ MOF/PAMAM-modified GCE. All concentrations are in µM. (n = 5).

Sample	Spiked		Found		Recovery (%)	
	Tramadol	Acetaminophen	Tramadol	Acetaminophen	Tramadol	Acetaminophen
Tramadol tablets	0	0	4.0 ± 0.05	-	-	-
	1.0	4.0	4.9 ± 0.04	4.1 ± 0.07	98.0	102.5
	2.0	6.0	6.2 ± 0.05	5.8 ± 0.05	103.3	96.7
	3.0	8.0	7.1 ± 0.03	7.9 ± 0.04	101.4	98.7
	4.0	10.0	7.9 ± 0.06	10.1 ± 0.04	98.7	101.0
Acetaminophen tablets	0	0	-	3.5 ± 0.05	-	-
	5.0	1.0	5.1 ± 0.05	4.4 ± 0.06	102.0	97.8
	7.0	2.0	6.8 ± 0.03	5.6 ± 0.03	97.1	101.8
	9.0	3.0	9.1 ± 0.04	6.4 ± 0.06	101.1	98.5
	11.0	4.0	10.9 ± 0.05	7.6 ± 0.04	99.1	101.3

## Data Availability

The data presented in this study are available on request from the corresponding authors.
